# The Chronic Kidney Disease Water Intake Trial: Protocol of a Randomized Controlled Trial

**DOI:** 10.1177/2054358117725106

**Published:** 2017-08-22

**Authors:** William F. Clark, Shih-Han Huang, Amit X. Garg, Kerri Gallo, Andrew A. House, Louise Moist, Matthew A. Weir, Jessica M. Sontrop

**Affiliations:** 1 Victoria Hospital, London, Ontario, Canada; 2 University Hospital, London, Ontario, Canada

**Keywords:** chronic kidney disease, copeptin, estimated glomerular filtration rate, randomized controlled trial, vasopressin, water intake

## Abstract

**Background:**

In observational studies, drinking more water associates with a slower rate of kidney function decline; whether the same is true in a randomized controlled trial is unknown.

**Objective:**

To examine the 1-year effect of a higher vs usual water intake on estimated glomerular filtration rate (eGFR) in patients with chronic kidney disease.

**Design:**

Parallel-group randomized controlled trial.

**Setting:**

Nine centers in Ontario, Canada. Enrollment and randomization occurred between May 2013 and May 2016; follow-up for the primary outcome will continue until June 2017.

**Participants:**

Adults (n = 631) with stage 3 chronic kidney disease (eGFR 30-60 mL/min/1.73 m^2^) and microalbuminuria.

**Intervention:**

The high water intake group was coached to increase their oral water intake by 1.0 to 1.5 L/day (depending on sex and weight), over and above usual consumed beverages, for a period of 1 year. The control group was coached to maintain their usual water intake during this time.

**Measures:**

Participants provided 24-hour urine samples at baseline and at 6 and 12 months after randomization; urine samples were analyzed for volume, creatinine, osmolality, and the albumin-to-creatinine ratio. Blood samples were obtained at baseline and at 3- to 6-month intervals after randomization, and analyzed for creatinine, copeptin, osmolality, and electrolytes. Other measures collected included health-related quality of life, blood pressure, body mass index, and diet.

**Primary outcome:**

The between-group change in eGFR from baseline (prerandomization) to 12 months after randomization.

**Secondary outcomes:**

Change in plasma copeptin concentration, 24-hour urine albumin-to-creatinine ratio, measured creatinine clearance, estimated 5-year risk of kidney failure (using the 4-variable Kidney Failure Risk Equation), and health-related quality of life.

**Planned analysis:**

The primary analysis will follow an intention-to-treat approach. The between-group change in eGFR will be compared using linear regression. Supplementary analyses will examine alternative definitions of eGFR change, including annual percentage change, rate of decline, and rapid decline (a *P* value <0.05 will be interpreted as statistically significant if there is concordance with the primary outcome).

**Trial Registration:**

This randomized controlled trial has been registered at www.clinicaltrials.gov; government identifier: NCT01766687.

## Abrégé

**Mise en contexte:**

Dans les études observationnelles, on a remarqué une association entre un apport hydrique accru et un ralentissement de la détérioration de la fonction rénale. Cependant, nous ignorions si ce phénomène s’observait également lors d’essais contrôlés à répartition aléatoire.

**Objectifs de l’étude:**

L’objectif était d’observer, sur une période d’un an, les effets d’un apport hydrique accru sur le débit de filtration glomérulaire estimé (DFGe) de patients atteints d’insuffisance rénale chronique (IRC) par rapport à l’apport hydrique habituel.

**Modèle de l’étude:**

Essai contrôlé à répartition aléatoire et à groupes parallèles.

**Cadre de l’étude:**

L’étude s’est tenue au sein de neuf centres hospitaliers de l’Ontario, au Canada. Le recrutement et la répartition des patients se sont échelonnés sur une période de trois ans, soit de mai 2013 à mai 2016. Le suivi des résultats primaires s’est poursuivi jusqu’en juin 2017.

**Participants:**

Un total de 631 adultes atteints d’insuffisance rénale de stade 3 (DFGe entre 30 et 60 mL/min/1,73 m^2^) et présentant une microalbuminurie.

**Méthodologie:**

Sur une période d’un an, nous avons demandé au groupe-test d’augmenter leur apport hydrique de 1 à 1,5 litre par jour, quantité établie selon le sexe et le poids du patient. Le groupe contrôle devait maintenir son apport hydrique au volume habituel.

**Mesures:**

Les participants devaient fournir des échantillons d’urine sur une période de 24 heures avant la répartition aléatoire, de même que six mois et douze mois après. Les échantillons d’urine ont été recueillis pour en mesurer le volume, le taux de créatinine et de copeptine, l’osmolarité et les électrolytes. Les autres paramètres analysés incluaient la pression sanguine, l’indice de masse corporelle (IMC), la diète et la qualité de vie générale des patients en considérant leur état de santé.

**Résultat primaire escompté:**

L’observation de variations entre les deux groupes au plan de la mesure de DFGe faite avant la répartition aléatoire et celle faite douze mois après.

**Résultats secondaires:**

Des variations dans la concentration plasmatique de copeptine, le ratio albumine-créatinine sur une période de 24 heures, la clairance de la créatinine, l’estimation du risque d’insuffisance rénale sur cinq (5) ans (en utilisant l’équation du risque d’insuffisance rénale à quatre variables) et la qualité de vie reliée à l’état de santé.

**Analyse prévue:**

L’analyse primaire suivra une approche d’analyse en intention de traiter. Les variations du DFGe entre les deux groupes seront comparées par analyse de covariance. Des analyses subséquentes se pencheront sur les différentes manières de définir les changements observés dans les mesures du DFGe, soit le pourcentage de la variation annuelle, le taux de déclin et le déclin rapide pour lequel une valeur de *p* plus faible que 0,05 sera interprétée comme étant significative statistiquement si elle concorde avec le résultat primaire.

**Enregistrement de l’essai clinique:**

Cet essai contrôlé à répartition aléatoire a été enregistré sur www.clinicaltrials.gov et le code d’identification du gouvernement est le NCT01766687.

## What was known before

- In observational studies, drinking more water associates with a slower rate of kidney function decline in adults.
- Whether supplemental water intake can preserve kidney function in patients with chronic kidney disease is unknown.

## What this adds

- A common question in a kidney clinic is “How much water should I drink?” The Chronic Kidney Disease Water Intake Trial will provide new information on the 1-year effect of a higher vs usual water intake on kidney function, plasma copeptin, microalbuminuria, and quality of life in adults with chronic kidney disease.

## Introduction

The purported benefits of drinking more water are largely untested, with many unfounded claims in the popular media.^1-3^ However, there is now a growing body of evidence on the specific effect of increased water intake on the kidney.^4^ Increased water intake has been shown to reduce the risk of kidney stones (a greater urine flow rate may lower the supersaturation of calcium oxalate, calcium phosphate, and uric acid) and lower the risk of stone formation,^5-7^ and guidelines recommend drinking water to achieve a urine output of 2.0 to 2.5 L/day to reduce the risk of stone recurrence.^8,9^ In polycystic kidney disease, there is some evidence that increased water intake may slow the growth of renal cysts via suppression of vasopressin;^10-12^ however, this has yet to be confirmed in a clinical trial.^13-15^ Most recently, chronic dehydration and volume depletion resulting from extreme occupational heat stress has been identified as a likely cause of an epidemic of chronic kidney disease in Central America.^16-20^ Scientists are now investigating the epidemiology of heat-stress nephropathy across the globe.^20-23^

The relationship between water intake and the estimated glomerular filtration rate (eGFR) has been investigated in several observational studies; however, results are mixed.^4^ In a retrospective analysis of adults patients with chronic kidney disease in the Modification of Diet in Renal Disease study, higher urine volumes were associated with a greater decline in eGFR; however, this association lost significance after controlling for diuretics and antihypertensive medication use.^24^ By contrast, in a prospective cohort study of adults free of chronic kidney disease at study entry, higher urine volumes at baseline were associated with a slower decline in eGFR over 7 years, and those with the largest urine volumes (>3L/day) were the least likely to demonstrate a rapid decline in eGFR (defined as a decline ≥5%/year).^25^ These results persisted after adjusting for age, gender, baseline eGFR, medication use for hypertension (including diuretics), proteinuria, diabetes, and cardiovascular disease. Interestingly, in studies examining the effects of plain water intake vs other fluids, increased intake of plain water is more often associated with a decreased risk of chronic kidney disease; however, increased intake of sweetened beverages is associated with an increased risk.^26-32^ Similarly, while the risk of kidney stone recurrence is reduced with greater intake of plain water,^8,9^ the risk of stone recurrence is increased with greater intake of sweetened beverages, possibly due to the high fructose content, which has been shown to increase the urinary excretion of calcium, oxalate, and uric acid.^33-36^

While these studies provide preliminary evidence that drinking more water may have a beneficial effect on kidney function, it remains unknown whether supplemental water intake can benefit patients with chronic kidney disease. Urine concentrating capacity is reduced as kidney function declines, and thus some patients with chronic kidney disease may have high 24-hour urine volumes while being slightly volume depleted, which makes it difficult to isolate causal effects in observational studies. To provide further insight into this question, we designed a randomized controlled trial to examine the effects of an increased vs usual water intake on kidney function in adults with chronic kidney disease—the Chronic Kidney Disease Water Intake Trial (WIT). In this parallel-group trial, participants randomized to the hydration group are coached to drink 1.0 to 1.5 L of water per day (depending on weight and sex), over and above their usual fluid intake, for 1 year. Participants randomized to the control group are asked to continue with their usual fluid intake during this time. The primary outcome of this trial is change in kidney function at 1 year. The trial design and methods were informed by a 6-week randomized pilot trial, which confirmed the safety and feasibility of asking adults with chronic kidney disease to increase their water intake by 1.0 to 1.5 L/day.^37,38^ The current protocol describes the objectives, methods, and analytic plan for the WIT main trial. To our knowledge, this will be the first clinical trial on record to test the impact of a sustained increase in water intake over 1 year in patients with chronic kidney disease.

**Primary objective**: To examine the effect of increased water intake over 1 year on change in eGFR among patients with chronic kidney disease.
**Secondary objectives**: To examine the effect of increased water intake over 1 year on change in plasma copeptin concentration, 24-hour urine albumin-to-creatinine ratio, measured creatinine clearance, the estimated 5-year risk of kidney failure (using the 4-variable Kidney Failure Risk Equation^39^), and health-related quality of life. We will also establish the safety of increased water intake in this population by monitoring for electrolyte disturbances, particularly hyponatremia.

## Methods

### Design, Setting, and Participants

WIT is a parallel-group, open-label, 9-center randomized controlled trial. Recruitment and randomization (detailed below) occurred between May 2013 and May 2016; follow-up for the primary outcome will continue until June 2017 (trial registration: www.clinicaltrials.gov; government identifier: NCT01766687). Ethics approval was obtained from Western University’s Health Sciences Research Ethics Board.

Adult patients with stage 3 chronic kidney disease were recruited from 9 chronic kidney disease clinics across Southwestern Ontario (Canada) in London (3 centers), Guelph (1 center), Hamilton (1 center), Oakville (2 centers), and Windsor (2 centers). The patient’s nephrologist invited interested potential participants to speak with a research assistant who explained the study and confirmed study eligibility (patient eligibility criteria are fully detailed in Table 1). All participants had to have stage 3 chronic kidney disease (eGFR 30-60 mL/min/1.73 m^2^) and microalbuminuria (defined as albumin/creatinine >2.8 mg/mmol if female, or >2.0 mg/mmol if male, or trace protein or greater from a random spot urine sample on an Albustix).^40^ Exclusion criteria (fully detailed in Table 1) included self-reported fluid intake ≥10 cups/day or a 24-hour urine volume ≥3L; a history of kidney stones in the past 5 years; or currently taking lithium, hydrochlorothiazide >25 mg/day, indapamide >2.5 mg/day, furosemide >40 mg/day, or metolazone >2.5 mg/day.

**Table 1.** Eligibility Criteria for the Chronic Kidney Disease Water Intake Trial.

Inclusion criteria
● Age between 18 and 80 years. ● Able to provide informed consent and willing to complete follow-up visits. ● Estimated glomerular filtration rate between 30 and 60 mL/min/1.73 m^2^. ● Trace protein or greater (Albustix) or urine albumin/creatinine ratio ≥2.8 mg/mmol (if female) or ≥2.0 mg/mmol (if male) from a random spot urine sample. ● Ability to read and speak English.
Exclusion criteria
● Self-reported fluid intake ≥10 cups/day or 24-hour urine volume ≥3 L. ● Enrolled in another randomized controlled trial that could influence the intervention, outcomes, or data collection of this trial (or previously enrolled in this trial). ● Received one or more dialysis treatments in the past month. ● Received a kidney transplant in past 6 months. ● Pregnant or breastfeeding. ● History of kidney stones in the past 5 years. ● Less than 2 years’ life expectancy. ● Serum sodium <130 mEq/L without suitable explanation. ● Serum calcium >2.6 mmol/L without suitable explanation. ● Currently taking hydrochlorothiazide >25 mg/day, indapamide >2.5 mg/day, furosemide >40 mg/day, or metolazone >2.5 mg/day. ● Currently taking lithium. ● Currently under fluid restriction (<1.5 L a day) for kidney disease, heart failure, or liver disease, AND meets any of the following criteria (1) end-stage disease (heart left ventricular ejection fraction <40%, NYHA class 3 or 4, or end-stage cirrhosis) or (2) any hospitalization for heart failure, ascites, and/or anasarca. ● Significant gastrointestinal disease (eg, inflammatory bowel disease or Crohn disease)

*Note.* NYHA = New York Heart Association.

After providing written informed consent, participants were asked to provide a prerandomization baseline 24-hour urine sample within 2 weeks (details provided in Data Collection and Measures section) to confirm that urine volume was below 3 L/day; participants were not randomized until their 24-hour urine samples were collected and analyzed.

### Randomized Allocation

Once study eligibility was confirmed, a research assistant contacted the participant by telephone to complete the randomization; concealed randomized allocation occurred by computer-generated randomization while the participant was on the phone. Participants were randomized (1:1) in random permuted blocks of varying sizes to the hydration group or the control group, stratified by center and gender. By necessity, research staff and participants were aware of the randomized group assignment; however, outcome assessors (technicians performing the laboratory measurements for the primary and secondary outcomes) are blinded to the random allocation, and the trial statistician will be blinded to patient allocation for the primary analysis.

### Intervention Group

Participants in the hydration group were coached to increase their oral water intake by 1.0 to 1.5 L/day (depending on sex and weight), over and above usual consumed beverages, for 1 year (detailed in Table 2). These water-intake levels were determined based on a review of prior literature^13,15,25,41-44^ and what we believed was safe in this population of patients with chronic kidney disease (considering that there is uncertainty in the benefit of additional water intake between 1.5 and 2.5 L/day, but risk of a total fluid intake below 1.0 L/day or greater than 4 L/day in kidney patients), and also demonstrated to be feasible in our pilot trial.^37,38^ A gradual increase in water intake was advised in the 2 weeks following randomization: during week 1, participants were instructed to consume 1 cup of water at breakfast, lunch, and dinner; and during week 2, the full amount according to weight and sex. Participants in the hydration group received reusable drinking containers and were mailed 20 vouchers per month; each voucher was redeemable for 1.5 L of bottled water.

**Table 2.** Hydration Intervention by Age and Sex.

Sex	Weight	Target water intake			
		Daily total (L/day)	Breakfast	Lunch	Dinner
Female	<70 kg	1.0	250 mL (1 cup)	500 mL (2 cups)	250 mL (1 cup)
	≥70 kg	1.25	250 mL (1 cup)	500 mL (2 cups)	500 mL (2 cups)
Male	<70 kg	1.25	250 mL (1 cup)	500 mL (2 cups)	500 mL (2 cups)
	≥70 kg	1.5	500 mL (2 cups)	500 mL (2 cups)	500 mL (2 cups)

### Control Group

Participants in the control group were coached to continue with their usual fluid intake or to decrease intake by 0.25 to 0.5 L/day (1-2 cups/day) if their baseline 24-hour urine volume was >1.5L/day and 24-hour urine osmolality was <500 mOsm/kg).^37^

We conducted monthly coaching with all participants (in both intervention and control groups) using interviewer-administered standardized surveys with questions on daily water intake. Coaching also included a discussion of urine color charts (showing the spectrum of dilute to concentrated urine), which were provided to all participants after randomization. Participants in the hydration group were encouraged to increase their water intake if daily intake fell below the target amount (based on sex and weight as shown in Table 2) or if they reported that their urine color was not light or clear (based on the urine color charts). Participants in the control group were asked to maintain as closely as possible the same amount ingested as reported at baseline. Coaching continued at monthly intervals for all participants for 12 months after randomization.

### Data Collection and Measures

The schedule of study measures is provided in Table 3. At baseline, a research assistant measured each participant’s height, weight, and blood pressure following a standardized protocol, and conducted an interviewer-administered health survey with questions on sociodemographics, medical history, smoking status, medication use, and fluid intake; this questionnaire also included 4 questions from the Kidney Disease Health-Related Quality of Life–Short Form (items 10, 17, 18, and 22), which relate to overall health, quality of life, and sleep.^45,46^ In addition, participants were asked about their appetite and frequency of urination during the day and night. The health survey (interviewer administered) was completed again over the phone or in person at 6 and 12 months after randomization. Weight and blood pressure were measured at the participants’ next kidney care clinic visit, approximately 8 to 12 months after enrollment. Participants also completed a 3-day diet record at baseline and again at 6 months, which they mailed back to the study center in a preaddressed postage-paid envelope; 3-day diet records were analyzed by The Food Processor (ESHA: Elizabeth Stewart Hands and Associates Research 2016 version 11.2) and a renal dietician provided individual consultations with participants to discuss their results on protein and sodium intake (participants were advised to follow a target intake of 0.8 g/kg/day of protein and 100 mmol/day of sodium).

**Table 3.** Schedule of Study Visits and Measures.

	Baseline^a^	Follow-up^b^					18-24 months
		3 weeks	3 months	6 months	9 months	12 months	
Survey							
Demographics	+						
Diet (3-day diet record)	+			+			
Health history	+			+		+	
Health-related quality of life	+			+		+	
Water survey	+		+	+	+		
Clinical							
Height (cm)	+						
Weight (Kg)	+				+^c^	+	
Waist circumference (cm)	+				+^c^	+	
Blood pressure (mm Hg)	+				+^c^	+	
Medications	+				+^c^	+	
Blood							
Blood sample	+	+	+	+	+	+	
Serum creatinine (μmol/L)	+	+	+	+	+	+	+^d^
Serum sodium (mmol/L)	+	+	+	+	+	+	
Urea (mmol/L)	+			+		+	
Osmolality (mOsm/kg)	+			+		+	
Copeptin (pmol/L)	+				+^c^		
Hematocrit (L/L)	+			+		+	
Cystatin C (mg/L)	+				+^c^		
HbA1c (%)	+			+		+	
Plasma samples for long-term storage	+						
Urine							
24-hour urine sample (L)	+			+		+	+^d^
Urine creatinine (mmol/d)	+			+		+	+^d^
Urine sodium (mmol/d)	+			+		+	
Urine potassium (mmol/d)	+			+		+	
Urea (mmol/d)	+			+		+	
Osmolality (mOsm/kg)	+			+		+	
Albumin (mg/d)	+			+		+	
Measured Creatinine clearance (mL/min/1.73 m^2^)	+			+		+	+^d^
Random spot urine sample	+						
Specific gravity (g)	+						
Osmolality (mOsm/kg)	+						

a Prerandomization.

b Time after randomization.

c While local labs are able to measure and process blood and urine samples, they do not measure weight, blood pressure, cystatin C, or copeptin; these measures will be obtained at the participants’ follow-up kidney care clinic visit (approximately 9 months after randomization).

d Posttrial data (18-24 months after randomization) will be obtained from participants’ medical charts where possible to reduce respondent burden. The “+” symbol indicates that the measure was collected at this time point.

Participants were instructed to collect a 24-hour urine sample (using a standard collection jug provided to them) within 2 weeks of enrollment and again at 6 and 12 months after randomization. Participants were able to deliver the 24-hour urine collection to a local laboratory (eg, LifeLabs, Gamma Dynacare, or Medical Laboratories of Windsor) or to the study center. The 24-hour urine samples were analyzed for the following measures: the albumin-to-creatinine ratio (measured using turbidimetric methods), sodium and potassium (measured using indirect potentiometry), urea (measured using enzymatic photometric methods), osmolality (measured using freezing point depression with an advanced instrument Micro-Osmometer), and creatinine clearance (body surface area corrected in mL/min/1.73 m^2^). In addition, to examine whether there was any lasting effect of the intervention after coaching stopped at 12 months, we collected data on 24-hour urine volume and creatinine from participant medical charts 18 to 24 months after randomization.

Participants provided nonfasting 10-mL blood samples at baseline, at 3 weeks after randomization, and then again at 3-month intervals after randomization until 12-months. Blood samples could be provided at the study research center or a local laboratory facility as described above. Serum creatinine was measured using the isotope dilution/mass spectroscopy–traceable enzymatic method, and eGFR was calculated using the Chronic Kidney Disease Epidemiology Collaboration (CKD-EPI) equation.^47^ Serum sodium was measured using indirect ion-selective electrodes. Other measures analyzed from blood samples at baseline and at 6 and 12 months after randomization included urea concentration (measured with enzymatic photometric methods), osmolality (measured by freezing point depression using an advanced instrument Micro-Osmometer), hematocrit (measured using Beckman Coulter automated cell counters), cystatin C (measured using immunonephelometry), glycated hemoglobin (HbA1c) (measured using nonporous ion exchange high-performance liquid chromatography), and copeptin (a glycosylated peptide that is coreleased with vasopressin from the hypothalamus^48,49^); copeptin was measured from nonfasting 150 µL blood samples, stored at −80°C, and analyzed in batches using the sandwich immunoluminometric assay (B.R.A.H.M.S. AG, Hennigsdorf/Berlin, Germany) as described by Morgenthaler et al.^48,50^ To examine whether there was any lasting effect of the intervention after coaching stopped at 12 months, we collected data on serum creatinine from participant medical charts 18 to 24 months after randomization.

### Outcomes

#### Primary outcome

The primary outcome of WIT is the change in eGFR from baseline (prerandomization) to 12 months after randomization.

#### Secondary outcomes

Key secondary outcomes include the following: Change in plasma copeptin concentration,^49,51-53^, 24-hour urine albuminuria,^54,55^ creatinine clearance, estimated 5-year risk of kidney failure (using the 4-variable Kidney Failure Risk Equation^39^), and health-related quality of life from baseline to 12 months after randomization. We will also establish the safety of increased water intake in this population by monitoring for electrolyte disturbances, particularly hyponatremia.

#### Posttrial outcomes

We will examine the between-group difference in 24-hour urine volume 18 to 24 months after randomization, and the between-group difference in eGFR change from baseline to 18 to 24 months after randomization.

### Analysis

We will use SAS version 9.3 (SAS Institute Inc, Cary, NC) for all statistical analysis.

The primary analysis will follow an intention-to-treat approach. The between-group difference on change in eGFR (calculated as eGFR at 12 months minus eGFR at baseline) will be analyzed using linear regression. The following prespecified covariates (measured at baseline) will be adjusted for in the primary analysis: age (in years), sex, obesity (body mass index ≥30 kg/m^2^), smoking status (current smoker: yes/no), presence of diabetes, albuminuria (mg/d), and use of any of the following medications: an angiotensin-converting enzyme inhibitor or angiotensin receptor blocker, diuretics, beta blockers or calcium channel blockers, and statins. We will include a missing data indicator value for each covariate.^56^ We expect that 12-month data on eGFR will be missing for <5% of participants due to death and <10% due to missing data or study withdrawal. For patients who were randomized but are missing a 12-month eGFR value, we will use recommended model-based multiple imputation methods to impute the final eGFR value.^57-59^ We will perform sensitivity analyses, including a complete-case analysis, to investigate whether conclusions are sensitive to assumptions about the missing-data mechanism.^57-59^

We will report the primary outcome as the absolute difference in 12-month eGFR change between randomized groups with a 95% confidence interval. Our current sample size of 631 randomized participants will provide 80% power to detect a difference of at least 1.8 mL/min/1.73 m^2^ between intervention groups (α = 0.05, independent samples *t* test, assuming a standard deviation of 8), which represents a small-to-moderate effect size. The primary analysis will be performed 12 months after the last patient has been randomized (date of final follow up expected in June 2017).

### Supporting Analyses

We will perform 3 additional analyses using alternative definitions of change in eGFR. For these analyses, a *P* value ≤ .05 will be interpreted as statistically significant provided there is concordance with the primary results.

1. Annual percentage change: We will calculate the annual percentage change as: [(final eGFR – baseline eGFR) / baseline eGFR] / [(final date – baseline date) / 365.21] × 100.^60^
2. Rate of eGFR change: We will estimate the rate of change in eGFR using a mixed-effects model with repeated measures.
3. Rapid eGFR decline: We will define rapid decline as an eGFR decline >20% from baseline, moderate decline as a decline from 5% to 20%, stable eGFR as a percent change within 5% of baseline values (reference), and increasing eGFR as a rise in eGFR ≥5% from baseline; these cut points were chosen based on a series of studies showing a U-shaped relationship between change in eGFR and risk of end-stage kidney disease and mortality.^61-63^ The risk of rapid decline will be estimated using a multinomial logistic regression model (reference group: stable eGFR).

We will report adherence to the allocated intervention in each of the 2 groups at 6 and 12 months of follow-up. We will also conduct a per-protocol analysis restricted to participants in the hydration group who maintained a 24-hour urine volume that was at least 0.5 L/day above their baseline value at 6 months and 12 months after randomization, and participants in the control group who maintained a 24-hour urine volume that was <0.5 L/day above their baseline assessment at each follow-up assessment.

### Secondary Outcomes

Changes in continuous secondary outcomes (plasma copeptin concentration, creatinine clearance, the 24-hour urine albuminuria, and the 5-year Kidney Failure Risk Equation developed by Tangri et al^39^) will be analyzed using linear regression as described above. Variables will first be assessed for normality and, if not normally distributed, will be transformed as appropriate. Finally, we will also examine the relationship between change in copeptin and kidney function, and examine whether this relationship differs between intervention groups. For all secondary analyses, including health-related quality of life, all between-group differences will be reported using 95% confidence intervals.

### Additional Analyses and Posttrial Outcomes

To examine whether there is any lasting effect of the intervention after coaching stopped at 12 months after randomization, we will compare the between-group difference in 24-hour urine volume at baseline and at 6, 12, and 24 months after randomization. We will also compare the between-group difference on change in eGFR between baseline and 24 months after randomization. Finally, we will examine the effect of the intervention on the 1-year change in mean arterial blood pressure, body mass index, and HbA1c.

### Safety and Data Monitoring

Trial conduct and safety was monitored by an independent Data Safety and Monitoring Board (DSMB). The DSMB received a descriptive summary of trial data and adverse events at 6- to 9-month intervals, with no planned interim statistical analysis of the primary or secondary outcomes. In terms of safety, a potential risk of increased water consumption among patients with chronic kidney disease is hyponatremia (serum sodium <130 mEq/L). Symptoms can range from mild to severe and include nausea and vomiting, headache, confusion, seizures, and decreased consciousness or coma.^64^ While no cases of hyponatremia were observed in our pilot study^37^ nor in previous intervention studies of increased water intake among elderly patients,^43^ data on serum sodium were monitored closely throughout the trial (blood samples were analyzed for serum sodium at baseline, at 3-weeks after randomization, and every 3 months thereafter). As well, the research coordinator inquired about symptoms of hyponatremia during monthly calls to review intervention adherence and tolerance.

## Discussion

To our knowledge, there has never been a randomized controlled trial in chronic kidney disease patients to determine whether increased hydration can preserve kidney function.^4^ To provide better estimates of the effect of water intake on kidney function, we designed a randomized controlled trial to examine the effect of increased water intake over 1 year on kidney function in adults with chronic kidney disease. The primary outcome will be change in eGFR at 1 year.

The worldwide prevalence of chronic kidney disease in adults is estimated to be 8% to 16%, affecting over 400 million people.^65,66^ Health care costs for chronic kidney disease exceed $20,000 per patient per year on average, and these costs more than triple when patients progress to kidney failure.^65,67,68^ Unfortunately, few effective low-cost interventions exist to reduce the risk of progressive kidney disease. There is increasing interest in whether vasopressin, an antidiuretic hormone, contributes to chronic kidney disease progression through its effects on renal hemodynamics and blood pressure.^4,69-73^ Vasopressin is the first hormone released during dehydration,^74^ and in experimental studies of rats, increased water intake was shown to suppress vasopressin, reduce proteinuria, and improve creatinine clearance.^71,72^ A recent 3-year trial demonstrated that tolvaptan, a vasopressin 2 receptor antagonist used to treat hyponatremia, was effective in slowing kidney function decline in patients with polycystic kidney disease; however, this treatment was associated with high toxicity which resulted in low adherence rates.^75^ Increased copeptin, a reliable marker of vasopressin, has also been linked to kidney function decline, micro-albuminuria, myocardial infarction, and end-stage kidney disease.^48,49, 51,76-78^ The effect of increased water intake on copeptin and the relationship between copeptin and kidney function will be examined in secondary analyses in this trial.

### Methods to Minimize Bias

Because our intervention of increased water intake is widely accessible, there is a potential for control-group contamination. To encourage adherence to the assigned intervention and to minimize cross-group contamination, monthly coaching was conducted with all participants for 12 months using a standardized survey with reference to quantity of water ingested relative to the target intake. Coaching also included a discussion of urine color charts (showing the spectrum of dilute to concentrated urine), which were provided to all participants. Intervention adherence will be assessed primarily by 24-hour urine collections (obtained at baseline and at 6 and 12 months after randomization, and analyzed by a local laboratory) and also by self-reported fluid intake (measured at three 3-month intervals during the trial). While we recognize that our study would benefit from other measures of hydration status, our primary outcome will be evaluated using an intention-to-treat analysis where participants are analyzed according to their original randomized group assignment. Nonetheless, at the last data review in December 2016, we observed significant separation between groups with respect to their average 24-hour urine volumes (mean 24-hour urine volume was 2.5 L/day and 1.8 L/day for the intervention and control groups, respectively; *P* < .001).

To examine whether there is any lasting effect of the intervention after coaching stopped at 12 months after randomization, we will compare the between-group difference in 24-hour urine volume at baseline and at 6, 12, and 24 months after randomization. We will also compare the between-group difference on change in eGFR between baseline and 24 months as a secondary outcome.

To avoid information bias, all participants, irrespective of randomized group assignment, will have the same measurement schedule, including lab testing. While it was not possible to blind participants to intervention allocation, technicians measuring the laboratory outcome variables were unaware of group allocation, and the biostatistician will be blinded to patient allocation for the primary analysis. To minimize missing data, research assistants followed up missing survey responses and discrepant data by telephone. As well, transportation assistance and home visits were offered to participants who were unable to complete the study requirements independently.

## Conclusion

Randomized trials provide some of the best estimates of treatment effects. The WIT is a parallel-group randomized controlled trial that will estimate the effect of increased water intake over 1 year on change in eGFR, plasma copeptin, microalbuminuria, and health-related quality of life in adults with chronic kidney disease. To our knowledge, this will be the first clinical trial of increased water intake in patients with chronic kidney disease. The significant separation between groups on 24-hour urine volume at 12 months after randomization means that we will have reliable estimates of the impact of increased water intake on change in eGFR and copeptin, and other indicators of kidney function. While we recognize that these are surrogate outcomes, the results of this trial will be important for understanding the relationship between increased water intake and kidney health, and may provide support for conducting a larger, definitive randomized controlled trial in the future.
